# Decades of emerging infectious disease, food safety, and antimicrobial resistance response in Vietnam: The role of One Health

**DOI:** 10.1016/j.onehlt.2021.100361

**Published:** 2021-12-14

**Authors:** Hung Nguyen-Viet, Steven Lam, Huong Nguyen-Mai, Dao Thu Trang, Vu Thi Phuong, Nguyen Do Anh Tuan, Dang Quang Tan, Nguyen Thu Thuy, Dang Thuy Linh, Phuc Pham-Duc

**Affiliations:** a International Livestock Research Institute, Hanoi, Vietnam and Nairobi, Kenya; b Center of Public Health and Ecosystem Research, Hanoi University of Public Health, Hanoi, Viet Nam; c Independent Consultant, Guelph, Canada; d FHI360**,** Viet Nam; e Vietnam One Health Partnership Secretariat, Hanoi, Viet Nam; f International Cooperation Department, Ministry of Agriculture and Rural Development, Hanoi, Viet Nam; g General Department of Preventive Medicine, Ministry of Health, Hanoi, Viet Nam; h Department of Animal Health, Ministry of Agriculture and Rural Development, Hanoi, Viet Nam; i Department of Environmental Quality Management, Vietnam Environment Administration, Ministry of Natural Resources and Environment, Hanoi, Viet Nam; j Vietnam One Health University Network, Hanoi, Viet Nam

**Keywords:** One Health, Ecohealth, Inter-sectoral collaboration, Government, Vietnam

## Abstract

Since facing outbreaks of severe acute respiratory syndrome and avian influenza A in 2003, Vietnam has increasingly applied a One Health approach to address emerging infectious diseases of animal origin. Here, we reflect on the challenges and opportunities of One Health in the context of zoonoses, food safety, and antimicrobial resistance, drawing on a stocktake of One Health training, policy, and research in Vietnam. We also report on the results of a virtual consultation workshop held on July 2021 with representatives from 32 institutions in Vietnam to explore future One Health directions. As Vietnam approaches nearly two decades of disease preparedness and response, we hope our experiences can provide practical insights to support countries in developing coordination mechanisms and moving the One Health agenda forward toward better public health outcomes.

## 1 Introduction

Since facing outbreaks of severe acute respiratory syndrome (SARS) and avian influenza A (H5N1) in 2003, Vietnam has increasingly applied a multi-sectoral approach to address emerging infectious diseases of animal origin [1,2]. This approach officially became a One Health approach in 2010 through the Hanoi Declaration [3]. Adopting the One Health approach provided a foundation for health, agriculture, environment, and other sectors to communicate, collaborate, and coordinate toward better public health outcomes [4].

As Vietnam approaches nearly two decades of disease preparedness and response, this study has two objectives: 1) to take stock of some of the achievements, constraints, and opportunities of One Health training, policy, and research in Vietnam, and 2) report on the prioritization of zoonotic and foodborne diseases and antimicrobial resistance issues of national significance to Vietnam. In doing so, we share lessons for countries in establishing One Health, coordinating mechanisms and moving the One Health agenda forward.

## 2 Methodology

One Health in Vietnam is primarily operationalized through the Vietnam One Health Partnership for Zoonoses (OHP). The Secretariat of OHP has been documenting One Health programs and policies in Vietnam, involving tracking publications, reports, and presentations related to disease prevention and control from conferences, workshops, and the literature. To take stock of One Health efforts in Vietnam, we drew on a review of the literature (both English and Vietnamese), a review of OHP documents, and a reflection of our own experiences working as One Health researchers and practitioners. We also analyzed major One Health projects implemented in Vietnam in the last 20 years.

To identify One Health priorities of national significance to Vietnam, a virtual workshop led by the International Livestock Research Institute was held in July 2021. Our goal was to explore the perspectives on future One Health directions from key actors including representatives from human, animal (livestock and wildlife), and environmental health sectors, along with government staff. A complete list of involved organizations is provided in [5].

## 3 One Health globally, regionally, nationally

One Health operates at all levels. At the global level, Vietnam has been supported by a wide range of One Health initiatives. For example, the tripartite collaboration between the Food and Agriculture Organization of the United Nations, the World Organization for Animal Health, the World Health Organization, and more recently the United Nations Environment Programme, concentrates on coordinating global activities to address health risks at the human-animal-ecosystems interfaces. Vietnam is also contributing to global initiatives. The country is co‑leading the Zoonotic Diseases Action Package of the Global Health Security Agenda, a 70-country effort to build capacities to prevent, detect, and respond to infectious disease threats.

As in East Africa, the Middle East, South Asia, and Europe, there exist regional One Health initiatives in Southeast Asia, such as the Association of Southeast Asian Nations, Asia-Pacific Economic Cooperation, and the Southeast Asia One Health University Network (SEAOHUN) [6]. For instance, SEAOHUN is a forum facilitating sharing, connection, and close cooperation in One Health education and research projects in Southeast Asia. Southeast Asia is a hotspot for emerging infectious diseases, which often emerge from intensified production of animal source foods, illegal trade of wildlife, and climate change [7]. The region has been at the center of global attention on such diseases since the pandemic-potential outbreak of H5N1 in the 2000s.

## 4 Key chronological events of national One Health policy adoption

**Fig. 1:**
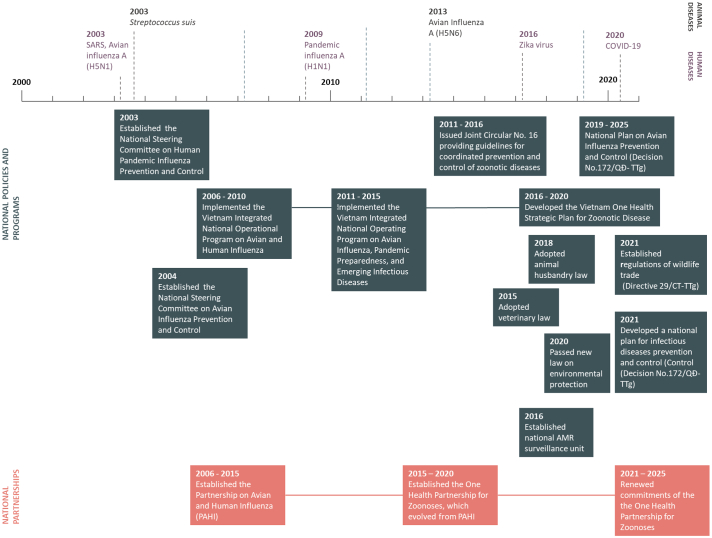
National responses to emerging infectious diseases in Vietnam from 2000 to 2021.

Over the past two decades, many emerging infectious diseases impacted Vietnam, including SARS (2003), H5N1 (2003), pandemic influenza A (H1N1) (2009), Zika virus (2016) [[8], [9], [10]], and COVID-19 (2020) (Fig. 1). Concurrently, diseases posing risks to both humans and animals such as *Streptococcus suis* (2003) and avian influenza A (H5N6) (2013) created substantial social and economic impacts. Prior to notable human and disease outbreaks in 2003, public health and veterinary sectors have already been working together to control zoonotic diseases in Vietnam such as rabies.

Vietnam's strategy toward disease prevention, response, and recovery has typically been “twofold, even threefold efforts” (*nỗ lực gấp đôi, gấp ba*), meaning putting in extra energy, time, and care [4]. Prompted by SARS, H5N, and H1N1, in the 2000s national steering committees have brought together key ministries and organizations for multi-sector action. These include the SARS Steering Committee (2003), the National Steering Committee on Avian Influenza Prevention and Control (2004), and the National Steering Committee on Human Pandemic Influenza Prevention and Control (2008) [4,11].

Under the direction of these steering committees, the Ministry of Health (MOH) and the Ministry of Agriculture and Rural Development (MARD) implemented joint strategic plans, such as the Vietnam Integrated National Operational Program on Avian and Human Influenza (2006–2010). Also in 2006, the Partnership on Avian and Human Influenza (PAHI) was established in 2006 with 26 national and international signatories to fill in gaps in disease prevention and control. In particular, PAHI mobilized sectors and disciplines to identify public health risks from H5N1 (initially) and other zoonotic diseases (later) and coordinate prevention and control actions.

Signaling Vietnam's intention to apply a One Health approach after the Hanoi Declaration, these steering committees also developed the Vietnam Integrated National Operational Program on Avian Influenza, Pandemic Preparedness, and Emerging Infectious Diseases (2011–2015) [3].

The Ministry of Natural Resources and Environment (MONRE) also supported the above plans. MONRE worked with MARD to guide the prevention and control of environmental pollution and remediation of environmental incidents. Specifically, guidance was provided on optimal measures to handle infected and dead animals which must be destroyed, and on treating waste from livestock production. MONRE also cooperated with MOH to revise regulations for the new Law on Environmental Protection (e.g. on medical waste management) [4].

In 2016, PAHI evolved into the Vietnam One Health Partnership for Zoonoses (OHP) (2016–2020) to strengthen One Health coordination in Vietnam and support the development and implementation of the Vietnam One Health Strategic Plan on Zoonotic Disease (2016–2020).

In early 2020, COVID-19 prompted the government to form a National Steering Committee for COVID-19 Prevention and Control, which is comprised of members from 23 ministries and agencies. Multi-sector steering committees were also established at provincial and local levels. Sectors are mobilizing resources for tasks relevant to their technical function. For example, MOH is primarily responsible for re-evaluating virus strains and identifying suitable countermeasures. MARD is ensuring agricultural products meet the needs of people nationwide. And MONRE is working to manage waste, especially medical waste in quarantine areas.

In early 2021, Vietnam launched the second phase of OHP (2021–2025), which was signed by three ministries (MARD, MOH, MONRE), with 28 national and international signatories, demonstrating Vietnam's ongoing commitment to One Health (Table 1). The partnership will continue to organize annual One Health Forums, establish different technical working groups based on focus areas, support the implementation of the One Health Strategic Plan, share information, support policy advocacy and strategic dialogue, mobilize resources, and build One Health capacity [2].

**Table 1:** List of signatories of the Vietnam One Health Partnership.

Group	Affiliations	Number of signatories
Ministries	- Ministry of Agriculture and Rural Development of the Socialist Republic of Vietnam - Ministry of Health - Ministry of Natural Resources and Environment	3
Vietnamese institutions	- Animal Husbandry Association of Vietnam - Center for Public Health and Ecosystem Research, Hanoi University of Public Health - Institute of Public Health and Capacity in Research and Development IPHCRD National Institute of Hygiene and Epidemiology - TAJ Vietnam JSC - Vietnam One Health University Network - Vietnam Public Health Association - Vietnam Veterinary Association	8
Embassies in Vietnam	- Australian Embassy - British Embassy - Denmark Embassy - French Embassy - Netherland Embassy - New Zealand Embassy - US Embassy	7
International organizations	- Agence Française de Développement - Alliance of Bioversity International and International Center for Tropical Agriculture - Centre de Coopération International En Recherche Agronomique pour le développement - Family Health International in Vietnam - Global Health Advocacy Incubator/Campaign for Tobacco-Free Kids - International Center for Antimicrobial Resistance Solutions - International Livestock Research Institute - Oxford University Clinical Research Unit - Vietnam - PATH Vietnam Country Office, - Research and Training Center for Community Development - The French National Research Institute for Sustainable Development - UN system - Wildlife Conservation Society, Vietnam Program	13

Resources have been shared via OHP's website (onehealth.org.vn), quarterly One Health newsletters, quarterly One Health Communication Network (OHCN) meetings, and bi-annual Research to Policy workshops. The MOH and MARD have cooperated to host several international and national conferences, including the International Ministerial Conference on Animal and Pandemic Influenza (2010), the National One Health Conference (2010, 2013, 2015), the One Health Forum (2018, 2019, 2020), the International GHSA Zoonotic Action Package Conference (2015, 2017), and the ASEAN-tripartite Rabies Elimination Strategy Conference (2018). That One Health issues know no borders make sharing information essential.

The many One Health policies make it difficult to attribute changes in disease patterns to a specific One Health policy. Often under-recognized yet important for Vietnam's success in controlling disease outbreaks is Vietnam's decisive and timely multi-sector action. Integrating One Health principles into policy allowed actors to do and achieve more. The resurgence of emerging infectious diseases had created a window for One Health policy. Without One Health policy, it is likely different sectors would not have been able to come together as effectively.

Key programmatic responses have been cross-sectoral and cross-disciplinary, supported by financial, policy, and technical assistance from UN agencies, international organizations, and national partners. Supported by the 10.13039/100004421World Bank, Vietnam was one of the first countries in Southeast Asia to conduct a health security financing assessment, which suggested Vietnam has made substantial progress toward implementing the One Health approach to strengthen collaboration and address zoonotic disease [12]. Programs such as USAID Wildlife Asia and USAID Strategies to Prevent Spillover are working with networks and partners in Vietnam to anticipate and mitigate risks of human and wildlife interactions; these programs are contributing to the mitigation of COVID-19, animal biosecurity threats, food safety issues, and other emerging challenges from human-animal interactions that require prevention and response [13].

International cooperation has also led to strengthened multi-sector collaboration. For example, a 2020 evaluation of the project ‘Strengthening capacity for the implementation of One Health in Viet Nam’ found the project was successful in promoting information sharing and multi-sectoral collaboration [14]. And in 2021, the ‘Emerging Pandemic Threats 2’ program was found to have largely achieved its objectives in terms of technical capacity development [15].

## 5 One Health education and training

The Vietnam One Health University Network (VOHUN), a member of OHP, is part of the regional SEAOHUN network, a university-led network of 24 universities and 30 faculties (public health, veterinary science, medicine, nursing, environment, and food technology) in Vietnam. Since its inception in 2011, VOHUN supported institutional changes in higher education by integrating competency-based One Health training and research into the curriculum [16]. Notably, VOHUN developed a field-based training course for health and veterinary professionals who are working on infectious disease prevention and control at provincial and district levels, promoting the interaction between universities, government agencies, professionals, and community members.

Universities are among key institutions supporting the management of emerging infectious diseases. For example, university members served on different taskforces by providing scientific evidence and proposing evidence-informed strategies. During COVID-19, VOHUN implemented a number of activities, including: a series of training workshops on Emergency Risk Communication, and Community Engagement for local health workers in Northern, Southern, Central, and Highland provinces in Vietnam; training needs assessment on laboratory bio-risk management; training of trainers course on biosecurity procedures for laboratory staff at Vietnamese universities; and, the establishment of the Student Outbreak Response Team. The One Health Communication Network and the Pandemic Prevention Taskforce are also bringing together human health, animal health, livestock, wildlife, and ecosystem health experts together from academic institutes in Vietnam.

## 6 One Health research

There is a tendency within the One Health community to focus on infectious diseases, especially zoonoses, with less consideration of other health issues that require multi-sectoral attention. Vietnam's first One Health Strategic Plan focused on seven focus areas: 1) Capacity building to implement the One Health approach, 2) Control of zoonotic diseases, 3) Control animal-originated pathogens, 4) Control zoonotic influenza virus with pandemic potential; 5) Rabies control, 6) Antimicrobial resistance (AMR) control, and 7) Control of other prioritized zoonotic diseases [4]. Vietnam is an active country in Southeast Asia promoting integrated approaches such as Ecohealth and One Health, bring together academic and non-academic partners to jointly implement transdisciplinary projects [17,18]. Below, we provide an overview of how several One Health issues were addressed in Vietnam. For a compendium of One Health programs in Vietnam, see [19].

### 6.1 Zoonotic diseases

Surveillance systems of zoonotic diseases operate at various scales depending on their pandemic potential. For example, active avian influenza virus surveillance is implemented in live bird markets in nearly 40% of the country [2], commonly detecting H5N1, H5N6, and H9. Vietnam also implements a longitudinal influenza surveillance network covering humans, livestock, and wildlife in three high-risk provinces. And for human populations only, the country has surveillance systems for severe acute respiratory infections, a leading cause of death among children worldwide. Additionally, with support from international partners, Vietnam has invested tremendously in rabies prevention and control. Specific strategies include rabies vaccination programs, management of dog populations, post-exposure prophylaxis for persons bitten by rabies-suspect dogs, and communication activities to raise community awareness [20]. Vietnam supports regional collaboration as the focal point in the development of the ASEAN Rabies Elimination Strategy as well as the action plan guiding its implementation [4]. Many zoonoses are circulating and causing considerable health and economic losses in Vietnam. Animal and human health sectors have been cooperating to address these less salient diseases. For instance, in 2016, MARD and MOH issued guidelines on preventing and controlling priority zoonoses, including anthrax, *Streptococcus suis,* and Leptospirosis (Circular 07/2016/TT-BNNPTNT).

### 6.2 Food safety

Food safety is a key area of One Health research and practice in Vietnam as it represents major concerns of the public and the government and therefore the government has several programs to control foodborne diseases [21]. In 2017, the Work Bank and partners at the request of the Vietnamese government published a comprehensive report on food safety challenges and opportunities in Vietnam and outlined key recommendations [22]. Although important, food-borne diseases receive limited attention from having a low potential to evolve into a pandemic. Nevertheless, programs are also implemented on the ground to manage these other priority diseases. For example, the SafePORK project (2017–2022) is developing market-based approaches to improve hygienic practices at slaughterhouses and wet markets. The World Health Organization is working with national organizations to strengthen lab-based surveillance systems for *Escherichia coli*, *Salmonella* spp., and *Shigella* in Hanoi, Dong Thap, and Vung Tau provinces [4]. A full list of food safety projects in Vietnam can be found in [4].

### 6.3 Antibiotic resistance

Vietnam was one of the first six countries in the Asia Pacific to develop and implement national action plans for combatting antimicrobial resistance (AMR) in the human health sector (2013) and livestock production and aquaculture (2017 and 2021). These plans have been supported by a national steering committee and its sub-committees from 2013 to 2020 and 2021–2025, with the strategic objective to “use fewer antimicrobials and use them more wisely” [4]*.* Vietnam's AMR response was propelled further by signing the Aide Memoire in 2015. This policy expresses the commitment of the MOH, MARD, Ministry of Industry and Trade (MOIT), MONRE, international organizations, and development partners to jointly implement national action plans across different sectors. Notable actions included implementing a national AMR surveillance unit in 2016 (Decision 6211/QD-BYT) and developing guidance for the use of antibiotics in hospitals (Circular 16/2018/TT-BYT), veterinary medicine (Circular 13/2016/TT-BNNPTNT), livestock feed (Circular 13/2020/ND-CP), and veterinary prescription (Circular 12/2020/TT-BNNPTNT). While these policies helped minimize pollution from antibiotic use, no dedicated policies were implemented to support the natural environment. Action against AMR is also supported by international aid programs; for example, the Fleming Fund, underpinned by £8.9 million, aims to improve laboratory capacity and surveillance of AMR in Vietnam across human health and animal health sectors.

### 6.4 Vietnam One Health lab and One Health site

A model of partnership between an international research institute and national partners was developed and shown as efficient in One Health research in Vietnam [23]. The International Livestock Research Institute, the National Institute of Veterinary Research, and the Hanoi University of Public Health have been working together since 2007 as a One Health lab on relevant topics to address intersectoral issues such as food safety, AMR, and zoonotic diseases in Vietnam. While One Health activities have been more focused on policies and trainings, research on One Health is less popular and therefore this One Health research partnership helped advance the knowledge of One Health in specific areas and generate local evidence on One Health. This lab implemented One Health projects, mobilized new resource, and translated research into practice. More than 40 peer-reviewed papers were published from this lab. The lab also worked together with VOHUN to develop a provincial-level One Health research site in Thai Nguyen Province where local partners and students can implement One Health case studies at the grassroot levels [24].

### 6.5 New initiatives on One Health

The Strategies to Prevent Spillover (STOP Spillover) project funded by USAID provides a critical opportunity to enhance global understanding of the complex drivers of viral spillover and address disease threats [25]. This five-year project will strengthen the capacity of 10 countries in Africa and Asia (including Vietnam) to: Monitor, analyze, and characterize the risk of priority zoonotic viruses spilling over from animals to people; Develop, test, and implement interventions and policies to reduce the risk of priority viral zoonotic spillover; and Mitigate the amplification and spread of priority zoonotic viral diseases if spillover occurs. In Vietnam, the STOP Spillover project is being implemented under the coordination of VOHUN in four target provinces, namely: Dong Nai; Dong Thap; Bac Giang; and Hanoi.

Another project titled Discovery & Exploration of Emerging Pathogens - Viral Zoonoses (DEEP VZN) plans to partner with up to 12 targeted countries in Africa, America and Asia, including Vietnam to carry out large-scale animal surveillance programs within their own countries using their own laboratory facilities [26]). The project will focus on finding previously unknown pathogens from three viral families that have a large potential for viral spillover from animals to humans, namely: coronaviruses, the family that includes SARS-CoV-2, the virus that causes COVID-19; filoviruses, such as the Ebola virus; and paramyxoviruses which includes the viruses that cause measles and Nipah. In Vietnam, the DEEP VZN project will be implemented under the management of PATH and FHI360.

### 6.6 Challenges and lessons learned

Vietnam's One Health efforts are supported by existing structures (formal policies, informal policies, partnership frameworks, international commitments), which were a product of several important starting conditions, namely growing awareness of One Health, history of inter-sectoral collaboration to address emerging infectious diseases, government leadership, and donor funding for actions toward global health security [2,4,27]. These structures provide the foundation for and define the boundaries of collaborative, coordinating, and communicative processes seen today.

Although structural elements are largely in place, many challenges remain for OH implementation in Vietnam. For instance, there needs to be deeper coordination with the environmental sector across all One Health foci, which tends to be neglected in the One Health triad (human-animal-environment) [28]. One limiting factor is the scarce research dedicated to environmental health and forestry protection in Vietnam [4]. To address this challenge, MONRE should play a larger role in the second phase of the One Health Partnership.

Resources are an important element for creating an enabling environment for One Health collaboration [29]. While international donors have contributed significant resources to developing national coordinating mechanisms, funding instability makes sustaining mechanisms beyond donor funding challenging [30]. Now, the government is trying to improve the situation by using counterpart funds and mobilizing different sources from international partners. However, if the national government was to provide funding, it would not be at the same level as donor funding [27]. Furthermore, because government funding for staff in Vietnam is low, staff turnover increases when international funding transfers to government funding, presenting risks to the sustainability of One Health strategies and outcomes. To build the One Health resilience and sustainability, it is important to engage the private sector and promote their investment.

While regulations are in place, the lack of capacity and human resources make enforcement challenging within the context of Vietnam's smallholder farming systems, especially in wildlife management [5]. There is a need for monitoring the adoption of 1) biosecurity procedures in livestock and wildlife farming; 2) food safety standards in slaughterhouses and markets; and, 3) AMR guidelines within farms. Strengthening capacity, offering incentives, and creating an enabling environment could support these efforts. In addition, collecting and making available examples, tools, and options for translating One Health from concept to practice in the field, particularly to the local context in different regions and populations, is essential.

The complex nature of disease emergence and control makes it important for Vietnam to continue developing its capacity to respond to current and future emerging infectious disease challenges. The OHP (previously PAHI) implemented since 2006 has been a major mechanism supporting One Health coordination. Though incredible progress has been made, the OHP still has room to grow, by improving the translation of research into policy, evolving from a coordinating mechanism into a sustainable One Health entity, promoting investments from the private sector, amplifying voices from civil society organizations, and achieving consensus on action plans moving forward.

Individual One Health focal areas in Vietnam also have unique challenges. For zoonoses, a major challenge is limited disease prioritization (e.g. only five prioritized zoonotic diseases out of 60 zoonoses in Vietnam) [5]. Importantly, most of the diseases from wildlife have pandemic potential but are not yet prioritized. Regarding food safety, there exist challenges in building infrastructure and applying food safety standards across smallholder farming value chains (e.g. farms, slaughterhouses, markets). As with food safety, inadequate value chain management also contributes to the inappropriate use of antibiotics, presenting further risks to health.

### 6.7 Moving the One Health agenda forward

Vietnam has taken important steps in developing and implementing One Health efforts for nearly two decades (2003−2021). Our virtual stakeholder workshop on 30 July 2021 brought together 90 participants from government ministries, universities, and national and international research organizations [5,31]. Participants shared their ideas on priority One Health issues of national importance to Vietnam, likely applicable to other countries as well, given many One Health challenges know no borders. Across the identified One Health priorities – zoonoses, food safety, and AMR – stakeholders emphasized, among other things, a need for engagement of policymakers and private sectors at the beginning of project development, climate change adaptation, and equity considerations (especially working with ethnic minorities) [5].

These perspectives build on previous efforts that highlighted gaps and opportunities for One Health in Vietnam. For example, a 2015 synthesis of One Health in Vietnam and other countries in Asia (e.g. China, Mongolia, and Bangladesh) identified the following issues, many of which still apply today: absence of incentives for inter-sectoral collaboration; ensuring donor goals are aligned with country goals; utilizing expertise within and outside of ministries effectively; ensuring appropriate research is conducted to understand disease problems, including use of participatory methods that meaningfully engages local stakeholders; economic assessments to demonstrate potential returns on investment; and ensuring there are sufficient resources available for One Health responses [32].

Vietnam's commitment to One Health is demonstrated by the Partnership Framework guiding One Health efforts from 2021 to 2025. The Partnership Framework is not a legally binding document, but rather reflects an inter-sectoral, multi-disciplinary approach under the leadership and guidance of the Government with active engagement of stakeholders at the provincial and central level, international partners, non-governmental organizations, academia, and civil societies. Partnership members are committed to sharing information for improved coordination within national One Health plans, contributing to One Health knowledge management and information sharing, improving One Health training activities, and supporting regional and international One Health cooperation. The Partnership Framework is also open to non-members who are willing to engage in the One Health activities.

The scope of our study was limited to insights into One Health efforts at the national level. Because organizations working to improve health at the local level also have rich experiences, future research should explore lessons from One Health initiatives at the community level.

## 7 Conclusion

The widespread impact of COVID-19 is a reminder of the need to work collectively across human health, animal health, and environmental sectors, not just during a crisis but after the crises are over. As the Vietnam case demonstrates, it is precisely in ‘peace time’ that coordinating mechanisms are strengthened through partnerships and joint actions. In sharing our experiences, challenges, and lessons learned, we hope other countries have gained valuable insights into preparing for and responding to One Health challenges.

## Declaration of Competing Interest

No conflict of interest is stated.
